# Effect of particle size of zeolite clay included in high-energy diets for feedlot lambs: Growth performance, dietary energy, carcass traits, and visceral mass

**DOI:** 10.5455/javar.2023.j703

**Published:** 2023-09-30

**Authors:** Beatriz I. Castro-Pérez, Jonathan Rodríguez-Vázquez, Alfredo Estrada-Angulo, Francisco G. Ríos-Rincón, Jesús D. Urías-Estrada, Elizama Ponce-Barraza, Alberto Barreras, Alejandro Plascencia

**Affiliations:** 1 Facultad de Medicina Veterinaria y Zootecnia, Universidad Autónoma de Sinaloa, Culiacán, México; 2 Instituto de Investigaciones en Ciencias Veterinarias, Universidad Autónoma de Baja California, Mexicali, México

**Keywords:** Zeolite, particle size, lambs, dietary energetic, visceral mass

## Abstract

**Objective:**

Several reports on the effects of zeolite (ZEO) inclusion in diets for feedlot lamb diets have indicated improvements in dietary energy efficiency and decreases in internal fat. Inclusion levels and the type of zeolite used have been the main focus of those reports. However, the possible effect of the zeolite particle size on the growth performance and carcass characteristics has not yet been investigated.

**Material and Methods:**

Forty-eight male intact Pelibuey × Katahdin lambs were fed for 67 days with a high-energy diet supplemented with ZEO as follows: 1) basal diet 0% ZEO (control); 2) basal diet 3% ZEO particle size 250 µM (sieve 60); 3) basal diet 3% ZEO particle size 149 µM (sieve 100); and 4) basal diet 3% ZEO particle size 74 µM (sieve 200). ZEO replaced corn grain and soybean meal in equal parts. At the end of feeding, lambs were slaughtered, and variables such as carcass characteristics and visceral mass were evaluated.

**Results:**

Particle size did not affect growth performance, carcass characteristics, or visceral mass. The inclusion of ZEO reduced 3.4% of dietary net energy in diets but did not affect dry matter intake or average daily gain, thus improving (*p *≤ 0.05) dietary energy efficiency by 2.9%. Except for an 18.9% reduction (*p* = 0.02) in visceral mass fat, ZEO did not affect other carcass characteristics or visceral organ mass.

**Conclusion:**

The particle size studied in the present experiment has not affected any of the variables of growth performance or carcass characteristics. ZEO inclusion could be a strategy to reduce the visceral fat in finishing lambs.

## Introduction

Clays, mainly zeolites (ZEO), are currently commonly used as feed ingredients in feedlot diets. Due to its molecular structure, ZEO has a great ion exchange ability and absorbability of several components, such as NH_3_-N [1,2]. In addition, ZEO alters the viscosity and pH of organic and inorganic fluids [3]. Those characteristics can change ruminal fermentation, absorption rate, and N retention in ruminants [4]. In such a manner, several reports on the effects of ZEO inclusion in diets for feedlot lambs indicated improvements in dietary energy efficiency and decreases in internal fat [5–7]. It is unclear what caused these improvements, but they might be attributable to improvements in ruminal N economy and increased digestible and metabolizable energy, primarily due to changes in fermentation and digestion [8]. Nevertheless, the capabilities of ZEO in trapping NH_3_-N and altering viscosity can be modified by the particle size of ZEO [9]. It is expected that larger particle sizes provide a greater surface area and therefore greater ion exchange capacity; moreover, particle size can change its properties on viscosity; therefore, particle size could affect the magnitude of the effects of zeolite when it is included in ruminant diets. However, the possible effect of the zeolite particle size on the growth performance and carcass characteristics has not yet been investigated. For this reason, this experiment aimed to evaluate the effect of the particle size of ZEO on growth performance, dietary energy, carcass traits, and visceral mass in hairy lambs fed a high-energy diet.

## Material and Methods

### Ethical approval

This experiment was conducted at the Universidad Autónoma of Sinaloa Feedlot Lamb Research Unit, located in Culiacán City, México (24° 46' 13" N and 107° 21' 14" W). Culiacán City is about 55 m above sea level and has a tropical climate. The average daily air temperature and relative humidity during the trial were 30.1°C and 38%, respectively. All animal management procedures were conducted according to the Mexico Federal Guidelines for Animal Use and Care and approved by the Ethics Committee of the Faculty of Veterinary Medicine and Zootechnics, Autonomous University of Sinaloa (protocol #03012023).

### Animals, treatments, and experimental design

Forty-eight Pelibuey × Katahdin [20.92 ± 3.29 kg initial body weight (BW)] intact male lambs were used in a 67-day growth-performance experiment to evaluate the treatment effects on growth performance, gain efficiency, and dietary energetics. Before the experiment, lambs were treated for endoparasites (Closantil, 5%, Chinoin Lab, México City, México) and injected with 1 × 10^6^ IU vitamin A (Synt-ADE^®^ , Fort Dodge, Animal Health, México). Lambs were fed the control diet (Table 1) for 4 weeks before the experimental period started. At the start of the experiment, lambs were individually weighed (electronic scale; TORREY TIL/S: 1072691, TORREY electronics Inc., Houston, TX), grouped by weight into six uniform weight blocks, and assigned to 24 pens (two lambs per pen). Individual pens were 6 m^2^ with overhead shade, automatic waterers, and 1 m fence-line feed bunks. Dietary treatments (Table 1) consisted of a corn-based finishing diet supplemented as follows: 1) basal diet 0% ZEO (control); 2) basal diet 3% ZEO particle size 250 µM (sieve 60); 3) basal diet 3% ZEO particle size 149 µM (sieve 100); and 4) basal diet 3% ZEO particle size 74 µM (sieve 200). ZEO replaced corn grain and soybean meal in equal parts. The ZEO source was calcic clinoptilolite with a purity of 92% (ZEO-SIL™; Grupo TCDN, Puebla, México); the level of ZEO used corresponded to the level for which better responses of growth performance and dietary energy in finishing lambs have been reported) [5–7]. The four dietary treatments were randomly assigned to pens within each weight block in a randomized complete block design. Lambs were allowed *ad libitum* access to both dietary treatments and clean water. Fresh feed was provided twice daily at 0800 and 1400 h at an approximately 40:60 proportion of the daily registered intake (on a feed basis). Daily feed allotments to each pen were adjusted to allow for approximately 5% residual feed remaining in the feed bunk during the morning feeding. Feed bunks were checked between 0740 and 0750 h each morning, and residual feed was collected and weighed to determine daily feed intake. Adjustments in daily feed delivery were made at the afternoon feeding. Lambs were individually weighed at the beginning of the trial and at the end of the experiment (day 67). The initial shrunk body weight (SBW) was determined as full body weight × 0.96 (adjustment for gastrointestinal fill). Upon completion of the study, all lambs were weighed following an 18-h fast (feed but not drinking water was withdrawn) to obtain the final SBW. Sampling of feed and refusals, as well as determination of dry matter, crude protein (CP), and neutral detergent fiber (NDF), were performed according to the techniques and procedures described by Urías-Estrada et al. [4].

### Calculations 

Estimates of average daily gain (ADG) and dietary net energy (NE) were based on the initial SBW and the final (d 67) fasted SBW. The ADG was computed by subtracting the initial SBW from the final SBW and dividing the result by the number of days on feed. Feed efficiency was computed as ADG/daily dry matter intake and expressed as a gain-to-feed ratio (GF). One approach for evaluating the efficiency of dietary energy utilization in growth-performance trials is the ratio of observed-to-expected DMI and observed-to-expected dietary NE. Based on the estimated NE concentration in the diet and growth performance measures, there is an expected energy intake. This estimation of expected DMI is performed based on the observed ADG, average SBW, and NE values of the diet (Table 1). Expected DMI, kg/d = (EM/EN_m_) + (EG/EN_g_), where EM (energy required for maintenance, Mcal/day) = 0.056 × SBW^0.75^, EG (energy gain, Mcal/day) = 0.276 × ADG× SBW^0.75^, and NE for maintenance (NE_m_) and NE for gain (NE_g_) are the NE contained in the experimental diets; those values were calculated based on the ingredient composition [10] of the basal diet (Table 1). The coefficient (0.276) was taken from the NRC [11], assuming a mature weight of 113 kg for Pelibuey × Katahdin male lambs. The observed dietary NE was calculated using the EM and EG values and the DMI observed during the experiment using the quadratic formula: *x* = (–b – √ b2 – 4ac)/ 2c, where: *x* = NE_m_, Mcal/kg, *a* = –0.41EM, *b* = 0.877 EM + 0.41 DMI + EG, and *c* = –0.877 DMI [7].

### Carcass data

All lambs were harvested on the same day. After humanitarian sacrifice, lambs were skinned, the gastrointestinal organs were separated and weighed, the omental and mesenteric fat were weighed, and the hot carcass weight (HCW) was registered. After carcasses (with kidneys and internal fat included) were chilled in a cooler at –2°C to 1°C for 24 h, the following measurements were obtained: 1) fat thickness perpendicular to the *m.* *longissimus thoracis* (LM), measured over the center of the ribeye between the 12th and 13th rib; 2) LM surface area, measured using a grid reading of the cross-sectional area of the ribeye between the 12th and 13th rib; and 3) kidney, pelvic, and heart fat (KPH). The KPH was manually removed from the carcass, weighed, and reported as a percentage of the cold carcass weight [12].

**Table 1.** Dietary composition of experimental diets fed to lambs.

Item	CON	Zeolite particle size (mesh size)^a^			
		60	100	200	
Ingredient composition, % of DM					
Corn grain cracked	57.00	55.50	55.50	55.50	
Sudangrass hay	12.00	12.00	12.00	12.00	
Distiller dry grain with solubles	8.50	8.50	8.50	8.50	
Soybean meal	10.00	8.50	8.50	8.50	
Cane molasses	8.00	8.00	8.00	8.00	
Tallow	2.00	2.00	2.00	2.00	
Zeolite	0.00	3.00	3.00	3.00	
Mineral-protein supplement ^b^	2.50	2.50	2.50	2.50	
Net energy concentration, Mcal/kg of DM ^c^					
EN_m_, Mcal/kg	2.07	2.00	2.00		2.00
EN_g_, Mcal/kg	1.41	1.36	1.36		1.36
Nutrient composition, % of DM^d^					
CP	14.77	13.97	13.97		13.97
NDF	19.47	19.09	19.09		19.09
Ether extract	5.58	5.49	5.49		5.49

DM = dry matter; Mcal = megacalorie; EN_m_ = Energy for maintenance; EN_g_ = Net energy for gain.

a The source of zeolite used was calcium clinoptilolite (Zeo-Sil, Grupo TCDN S.A., Puebla, Mexico) which contains a purity of 97% of the mineral. Particle sizes were 250, 149, and 74 microns, equivalent to 60, 100, and 200 mesh).

b Mineral-protein supplement contained (%): Ca, 13.58%; P, 0.40%; CP 50.5%; NaCl,18.0%, Mg 1.0%; K, 0.71%; Co, 5.6 ppm; Cu, 20.4 ppm; Mn, 1,674 ppm; Fe, 2759 ppm; and Zn, 2900 ppm.

c Based on tabular NE values for individual feed ingredients (NRC 2007).

d Dietary composition was determined by analyzing subsamples collected and composited throughout the experiment. Accuracy was ensured by adequate replication with acceptance of mean values that were within 5% of each other.

### Visceral mass

Visceral organs and empty body weight (EBW) estimation were processed following the method described by Coronel-Burgos et al. [5]. All tissue weights are reported on a fresh tissue basis. Organ mass is expressed as grams of fresh tissue per kilogram of final EBW, where final EBW represents the final full live weight minus the total digesta weight. The full visceral mass was calculated by the summation of all visceral components (stomach complex + small intestine + large intestine + liver + lungs + heart), including digesta. The stomach complex was calculated as the digesta-free sum of the weights of the rumen, reticulum, omasum, and abomasum.

### Statistical analysis

Growth performance data (ADG, DMI, gain efficiency, and dietary energetics) were analyzed as a randomized complete block design, using initial weight as the blocking criterion and a pen as the experimental unit [13]. Treatment effects were tested using orthogonal polynomials unequally spaced. In addition, means separations were performed using the “honestly significant difference test” (Tukey’s HSD test). Contrasts are considered significant when the *p*-value is ≤0.05.

## Results

Since the cubic effect was insignificant (*p *> 0.05) in any of the variables evaluated, the significance value for that component was omitted from the tables. The average daily intake of ZEO treatments averaged 32.7 gm/lamb. Particle size did not affect (*p *≥ 0.05) growth performance, carcass characteristics, or visceral mass (Tables 2). The inclusion of ZEO reduced 3.4% of dietary NE in diets but did not affect (*p *≥ 0.75) DMI or ADG, thus slightly improving (*p *≤ 0.05) dietary energy efficiency by 2.9% (). Except for an 18.9% reduction (*p *= 0.02) in visceral mass fat, ZEO did not affect other carcass characteristics or visceral organ mass ( and ).

## Discussion

Zeolite particle size has resulted in changes in its capabilities regarding molecule capture, buffering capacity, and its effects on the viscosity of fluids when tested in closed systems [3,9]. As was previously exposed, there is no information about the effects of ZEO particle size on animal performance. The absence of the effects on particle size observed in the present experiment could be due to insufficient ZEO concentration in the rumen environment or to the dynamics of the gastrointestinal tract system. In closed systems, there is a higher probability of interaction between ZEO and the molecules with which it has affinity, while in dynamic systems, this probability is decreased. Particle size is related to density; a reduction in particle size increases particle density (mass or volume) so that a smaller particle size has greater specific gravity and theoretically could escape from the rumen in less time [14]. Thus, we hypothesized that the smaller particle size would affect in a lesser manner the changes mainly regarding rumen fermentation and NH_3_-N retention, reducing the positive effects of ZEO on dietary energy improvement and reduction of carcass fat. Nevertheless, at the 3% supplementation level, ZEO particle size did not affect any measured variables. Further research is needed about the possible effects of particle size at higher levels of supplementation (i.e., 5%) on rumen fermentation, digestion, and performance in feedlot lambs.

Characteristically, ZEO inclusion from 2 up to 4.5% in finishing diets for lambs has reduced energy intake without affecting DM intake or ADG. In such a manner, under an energetic perspective approach, ZEO has consistently improved the efficiency of diet energy utilization [6,7,15]. This result is due to the “dilution effect” caused by the inclusion of inorganic supplements in diets (that do not themselves contribute energy), which is because the natural energy-rich ingredients in feed have been replaced with inorganic supplements. In this way, the magnitude of energy dilution will be directly related to the inclusion level of ZEO and the type of ingredient replaced.

**Table 2.** Effect of particle size of zeolite included at 3% level replacing corn grain and soybean meal in equal parts in finishing diet for feedlot lambs.

Item		Zeolite particle size (mesh size)			SEM	*p*-value		
	CON	M60	M100	M200		CON versus ZEOL	Linear	Quadratic
Days on test	67	67	67	67				
Pen replicates	6	6	6	6				
Live weight, kg ^a^								
Initial	20.87	20.99	20.94	20.92	0.106	0.51	0.75	0.45
Final	40.04	39.54	39.97	39.83	0.965	0.82	0.96	0.70
Average daily gain, kg	0.286	0.277	0.284	0.282	0.014	0.75	0.99	0.62
Dry matter intake, kg/day	1.097	1.081	1.103	1.089	0.047	0.91	0.87	0.76
Gain to feed, kg/kg	0.264	0.257	0.258	0.261	0.003	0.14	0.30	0.17
Observed dietary Net energy, Mcal/kg								
Maintenance	2.05	2.02	2.02	2.04	0.016	0.23	0.29	0.34
Gain	1.39	1.36	1.36	1.38	0.014	0.23	0.29	0.34
Observed to expected dietary Net energy ratio								
Maintenance	0.99	1.01	1.01	1.02	0.008	0.03	0.15	0.14
Gain	0.98	1.00	1.00	1.01	0.009	0.04	0.32	0.30
Observed to expected daily dry matter intake	1.01	0.99	0.99	0.98	0.009	0.05	0.26	0.24

DM = dry matter.

a Initial live weight (LW) was reduced by 4% to adjust for the gastrointestinal fill. Final LW was obtained following an 18-h fast without access to feed (access to drinking water was not restricted).

**Table 3.** Effect of particle size of zeolite included at 3% level replacing corn grain and soybean meal in equal parts in finishing diet for feedlot lambs.

Item	Zeolite particle size (mesh size)				SEM	*p*-value		
	CON	M60	M100	M200		CON versus ZEOL	Linear	Quadratic
HCW, kg	22.93	22.19	22.93	22.60	0.542	0.58	0.78	0.30
Dressing percentage	57.22	56.15	57.30	56.73	0.543	0.44	0.63	0.14
Cold carcass weight	22.77	22.05	22.87	22.47	0.569	0.65	0.70	0.32
LM area, cm	16.96	16.88	17.23	17.29	0.371	0.41	0.13	0.83
Back fat thickness, mm	1.47	1.53	1.48	1.76	0.245	0.66	0.99	0.86
KPH, %	2.15	1.74	2.00	1.97	0.129	0.11	0.90	0.17

LM =* m. longissimus thoracis*; KPH = kidney, pelvic, and heart fat.

**Table 4.** Effect of particle size of zeolite included at 3% level replacing corn grain and soybean meal in equal parts in finishing diet for feedlot lambs.

Item	Zeolite particle size (mesh size)				SEM	*p*-value		
	CON	M60	M100	M200		CON versus ZEOL	Linear	Quadratic
EBW (percentage of full weight)^b^	91.38	89.80	90.53	90.88	0.705	0.24	0.62	0.16
Full viscera, kg	8.59	8.98	8.80	9.14	0.226	0.16	0.70	0.26
GIT fill, kg	4.06	4.68	4.48	4.52	0.265	0.13	0.44	0.16
Organs (g/kg EBW)								
Stomach complex	26.04	26.00	25.38	25.73	1.112	0.73	0.65	0.91
Intestines	38.11	37.76	35.52	35.05	1.416	0.24	0.18	0.83
Heart/lungs	22.87	22.32	21.78	21.22	1.068	0.39	0.53	0.85
Liver	15.41	15.41	15.20	14.73	0.745	0.73	0.83	0.95
Kidney	2.45	2.33	2.30	2.24	0.121	0.27	0.43	0.64
Omental fat	18.21	14.91	15.43	14.63	1.114	0.01	0.41	0.16
Mesenteric fat	6.95	4.90	5.43	6.43	0.540	0.09	0.08	0.96
Visceral fat	25.16	19.81	20.86	20.49	1.285	0.02	0.46	0.22

EBW = Empty body weight; GIT = gastrointestinal tract.

By estimating dietary NE by measuring growth performance, we can gain insights into how additives (or other factors) affect the efficiency of dietary energy use [16]. Based on tables of feedstuff standards [10] and observed DMI during the trial, the observed-to-expected dietary NE ratio was 1.00, indicating that performance was consistent with the dietary NE values. A ratio greater than 1.00 indicates greater dietary energy use efficiency, whereas a ratio below 1.00 indicates lower dietary energy use. Lambs that received ZEO showed a slight (1.02), but significant (*p *= 0.04) increase in dietary energy use, while the control group was in close agreement with the expected (0.99). It has previously been noted that supplemental zeolite may enhance ruminal N economy [17], digestible energy [18], and metabolizable energy through modifications in the proportion of VFA in the rumen [19]. Moreover, a recent report indicated that ZEO enhanced starch utilization in the diet [4]. Those effects deal with growth performance improvements or efficiency in dietary energy utilization. Nevertheless, the mechanism underlying the improvement in dietary energy when ZEO was included in the diets needs to be elucidated in future studies. Even so, the improvement in dietary NE efficiency observed in the present experiment was lower than the average improvement reported previously. Urías-Estrada et al. [8] analyzed a series of 10 experiments where ZEO was included in fattening diets for feedlot cattle and feedlot lambs. An average increase of 4.5% in dietary energy efficiency was estimated when zeolite was included in a range of 2% to 5%. This value is significantly higher than the 2.4% estimated in this experiment. The reason for the lower relative improvement in dietary energy observed in this experiment for ZEO treatments is uncertain. Factors such as the associative effects of ingredients, the level of inclusion, and the strategy of replacements could affect the magnitude of the response to dietary energy use when ZEO is included in diets. In this case, ZEO inclusion replaced cracked corn grain and soybean meal in equal parts (1.5% each), reducing diet NE by 3.4% (2.07 *vs.* 2.00 Mcal NE_m_/kgdiet).

As it happened in the present experiment, when ZEO is included at levels equal to or below 3% in diets, carcass characteristics are generally not affected [5,6,20]. Decreases in dressing percentage have been reported in feedlot cattle when ZEO is included at 5% in the diet [21]. The decreases in HCW or dressing percentage when high levels of ZEO are included in diets could be explained by the reduction in energy intake in cattle supplemented with ZEO during a long-term period of feeding and by a possible remnant of the clay in the gastrointestinal tract (GIT) at the time of slaughter. Considering that ZEO is not absorbed during its transit in GIT, it is expected that some remnants of ZEO will remain before sacrifice. In this sense, we noted an average numerical increase in GIT fill of almost 0.5 kg (*p *= 0.13) for lambs fed with ZEO. Urías-Estrada et al. [4] estimated a relationship intake/excretion for ZEO of 0.74%, thus remaining at 26% ZEO for a longer time in GIT.

In accordance with previous reports [5,6], the inclusion of ZEO in the finishing phase of fattening reduced the content of visceral fat. The reduction of visceral fat has been explained mainly by energy dilution resulting from the ingredient replacement by ZEO and by changes in ruminal fermentation by ZEO, which can decrease ruminal acetate [4,19]. Those effects can promote the reduction of visceral fat depots [22,23].

The absence of the effects of the particle size observed in the present experiment could be due to insufficient ZEO concentration in the rumen environment or to the dynamics of the gastrointestinal tract system. Further research is needed about the possible effects of particle size at higher levels of supplementation (i.e., 5%) on rumen fermentation, digestion, and performance in feedlot lambs.

## Conclusion

The particle size studied in the present experiment has not affected any of the variables of growth performance or carcass characteristics. On the other hand, ZEO inclusion could be a strategy to reduce the visceral fat in finishing lambs without affecting growth performance.

## List of Abbreviations

ADG, average daily gain; CP, crude protein; EBW, empty body weight; HCW, hot carcass weight; KPH, kidney, pelvic, and heart fat; LM, *m.* *longissimus thoracis*; LW, live weight; NDF, neutral detergent fiber; NE_g_, net energy for gain; and NE_m_, net energy for maintenance.
